# Differences in Total Brain Volume between Sexes in a Cognitively Unimpaired Elderly Population

**DOI:** 10.6061/clinics/2020/e2245

**Published:** 2020-11-26

**Authors:** Marina Buchpiguel, Pedro Rosa, Paula Squarzoni, Fabio L.S. Duran, Jaqueline H. Tamashiro-Duran, Claudia C. Leite, Paulo Lotufo, Marcia Scazufca, Tania C.T.F. Alves, Geraldo F. Busatto

**Affiliations:** I Departamento e Instituto de Psiquiatria, Faculdade de Medicina (FMUSP), Universidade de Sao Paulo, Sao Paulo, SP, BR.; II Laboratorio Neuro-Imagem em Psiquiatria (LIM/21), Departamento e Instituto de Psiquiatria, Faculdade de Medicina (FMUSP), Universidade de Sao Paulo, Sao Paulo, SP, BR.; III Unidade de Pesquisa Clinica e Epidemiologia, Faculdade de Medicina (FMUSP), Universidade de Sao Paulo, Sao Paulo, SP, BR.; IV Departamento de Radiologia, Faculdade de Medicina (FMUSP), Universidade de Sao Paulo, Sao Paulo, SP, BR.; V Escola de Ciencias Medicas, Santa Casa de Sao Paulo, Sao Paulo SP, BR.

**Keywords:** Brain, Sexual Dimorphism, Magnetic Resonance Imaging, Aging, Gray Matter, White Matter

## Abstract

**OBJECTIVES:**

Although a large number of studies have shown brain volumetric differences between men and women, only a few investigations have analyzed brain tissue volumes in representative samples of the general elderly population. We investigated differences in gray matter (GM) volumes, white matter (WM) volumes, and intracranial volumes (ICVs) between the sexes in individuals older than 66 years using structural magnetic resonance imaging (MRI).

**METHODS:**

Using FreeSurfer version 5.3, we obtained the ICVs and GM and WM volumes from the MRI datasets of 84 men and 92 women. To correct for interindividual variations in ICV, GM and WM volumes were adjusted with a method using the residuals of a least-square-derived linear regression between raw volumes and ICVs. We then performed an analysis of covariance comparing men and women, including age and years of schooling as confounding factors.

**RESULTS:**

Women had a lower socioeconomic status overall and fewer years of schooling than men. The comparison of unadjusted brain volumes showed larger GM and WM volumes in men. After the ICV correction, the adjusted volumes of GM and WM were larger in women.

**CONCLUSION:**

After the ICV correction and taking into account differences in socioeconomic status and years of schooling, our results confirm previous findings of proportionally larger GM in women, as well as larger WM volumes. These results in an elderly population indicate that brain volumetric differences between sexes persist throughout the aging process. Additional studies combining MRI and other biomarkers to identify the hormonal and molecular bases influencing such differences are warranted.

## INTRODUCTION

Several studies have documented the brain’s anatomical differences across various age groups and sexes and multiple theories aim to explain the cause of these disparities. An example is to attribute these differences to hormones, for instance, the action of estrogen on synaptogenesis and the effects of the absence of this hormone during menopause (1,2). There is no consensus, however, regarding whether the differences between sexes are due to an actual disparity in the size of the brain structures or if this is merely a reflection of the varied cranial volumes among individuals (3). Regardless, it is known that the brain ages asymmetrically between men and women (4). The distinction of the volume of neuroanatomical structures in elderly participants of both sexes without any neurodegenerative diseases can help build a better understanding of the healthy aging process as well as a clearer understanding of the possible source of neuroanatomical differences between sexes.

The estimated volume of the cranial cavity, called the intracranial volume (ICV), is one of the main variables studied in the neuroanatomical variations between men and women (5). Overall, the literature consistently reports larger ICVs in men than in women(6-8). Total volumes of gray matter (GM) and white matter (WM) are also usually larger in men (6,9). Nonetheless, there is no consensus on the effects of sexual dimorphism on these brain volumes, as a larger GM or WM volume in men can be a mere reflection of larger ICV (3). Therefore, it is necessary to correct the total volumes of WM and GM to increase the validity of this type of investigation (10).

Studies that have compared brain volumes between sexes after ICV correction methods have yielded mixed results. There is a tendency for larger GM volumes in women, while the results regarding WM volumes have been more varied (6,9,11-14). These variations may be influenced by demographic variables, such as age (6) and years of schooling (15-18).

In the present study, neuroanatomical differences between sexes were investigated in a group of 176 individuals without neurodegenerative diseases (84 men and 92 women) residing in São Paulo, Brazil, and above the age of 66 years. We analyzed the values of the ICV, total GM volume, and WM volume without any adjustments. The volumes of GM and WM were then analyzed after using an ICV correction named as the residual method, which has been validated in recent studies (3,10,19). Furthermore, we evaluated the relationship between total brain volume and age (due to the critical relevance of age on brain volume variations in healthy aging individuals) (6) and the relationship between total brain volume and years of schooling, given that our sample included a considerable number of individuals with low levels of education, in contrast to most studies reported so far.

According to the present literature, we expected to find larger non-adjusted brain volumes in men. However, after correction for ICV, we expected to find significantly larger volumes of GM in women. Regarding WM, due to the inconsistent findings in the literature, we did not establish an *a priori* hypothesis. Finally, we predicted that fewer years of schooling would be associated with smaller brain volumes, both in men and women.

## MATERIAL AND METHODS

### Participants

We analyzed MRI scans of 176 individuals (84 men and 92 women) between 66 and 80 years of age. The group of elderly individuals described herein was drawn from a population-based sample recruited for a morphometric MRI study evaluating the association between cardiovascular risk factors and GM volumes in elderly individuals aged 65-75 years from a circumscribed, socioeconomically disadvantaged area of São Paulo, Brazil (São Paulo Ageing & Health - SPAH study). We also gathered information about the participants’ sex, socioeconomic status, and years of schooling.

All participants were healthy, with no signs of dementia or other neurodegenerative diseases. Further details regarding inclusion and exclusion criteria as well as the clinical evaluation of patients, can be found in Squarzoni 2016 (20).

### Data Acquisition and MRI Processing

All participants in the present study underwent a structural MRI scan, following the same imaging acquisition protocol, and were examined using the same equipment (Squarzoni 2018) (21). The structural MRI data were acquired from the Magnetic Resonance Sector of the Radiology Institute (InRad) at the University of São Paulo’s Medicine Faculty Clinics Hospital (HC-FMUSP) using a 1.5 T General Electric Signa LX CVi scanner (Milwaukee, WI, USA) with a quadrature transmit/receive head coil with AC-PC alignment using the following acquisition protocol: (a) axial T2 multiecho sequence of 100 slices with repetition time (TR) of 2800 ms and echo time (TE) between 25 and 250 ms with a total of 10 echo trains per excitation, flip angle (FA) of 90 degrees, 240 mm field of view (FOV), 5 mm slice thickness, number of excitations (NEX) of 1, and an acquisition matrix of 256×192 mm; (b) T2 axial FLAIR sequence of 20 slices with TR of 10002 ms and TE of 109.44 ms, FA of 90 degrees, 240 mm FOV, 5 mm slice thickness, 6.5 mm spacing between slices, NEX of 1, and an acquisition matrix of 256×192 mm; (c) spoiled gradient echo (SPGR) sequence of 124 contiguous slices with TR of 12.1 ms and TE of 4.2 ms, FA of 15 degrees, 240 mm FOV, 1.5 mm slice thickness, and an acquisition matrix of 256×192 mm; and (d) axial T2 sequence of 15 slices with TR of 4200 ms and TE of 12.5 ms, FA of 90 degrees, 280 mm FOV, 5 mm slice thickness, 6.5 mm spacing between slices, and an acquisition matrix of 256×192 mm.

### Generation of Intracranial Volume, WM Volume, and GM Volume

From the SPGR sequence described above, we extracted the values of the ICV, GM volume, and WM volume. The data were analyzed with FreeSurfer version 5.3 (http://surfer.nmr.mgh.harvard.edu/).

As described by Buckner et al. (22), FreeSurfer calculates the ICV automatically by dividing a predetermined constant by the Atlas Scaling Factor (ASF), a factor by which the individual MRI datasets of each participant are scaled linearly in size to align with a cerebral atlas (MNI305) created by merging various brain templates from participants with a wide age span. Buckner et al. (22) demonstrated that the use of this ASF method was reliable and consistent with manual measurements of ICV. The volumes were obtained by running a command called “recon-all-autorecon1,” which automatically called mri_segstats, to generate the ICV measurements. (23). The same command was used to obtain the WM and GM volumes, estimated using an ASF that spatially conformed the datasets of each individual to a template built using the same principles described for the ICV methodology.

### ICV Correction Methods for the Total Volumes of WM and GM

For the correction of total WM and GM volumes in each participant, we utilized the residual method, which is executed by running a linear regression between the value of the ICV and the neuroanatomical volume in question, WM or GM. Subsequently, we obtained the beta values (β) for the WM and GM volumes, after which we used the following mathematical formula:

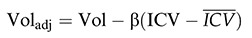



In this formula, Vol_adj_ is the ICV-corrected volume of the GM or WM, Vol is its original volume, without correction, β is the inclination of the linear regression, ICV is the intracranial volume of each individual, and 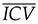 is the mean of the ICV of all participants (3,12).

The residual method was successfully implemented because our sample consisted of very similar groups of men and women, with comparable sizes and demographic variables (12).

### Statistical Analyses

Statistical analysis was carried out using the Statistical Package for Social Sciences (SPSS) IBM Statistics for Windows version 25.0 (SPSS, Chicago, IL, USA). The significance threshold was set at 0.05.

First, we ran an independent sample t-test to compare age and years of schooling between sexes. The qui-squared test was used to describe the differences in the socioeconomic status among men and women in the study sample.

Furthermore, we carried out an analysis of covariance (ANCOVA) comparing GM volume, WM volume, and ICV separately between sexes. Since we decided to use the residual ICV correction method, we needed to obtain the beta values for GM and WM volumes, both of which had very similar results (0.849 and 0.867, respectively). The GM and WM volumes were adjusted after we used the formula described in the previous section “ICV Correction Methods for the Total Volumes of WM and GM” with which we were able to generate the value of Vol_adj._ This new volume (Vol_adj_) was then used in the ANCOVA test as a covariable.

Subsequently, we ran another ANCOVA, comparing the ICV, GM volume, and WM volume between sexes, after including age and years of schooling as confounding variables. The values of Vol_adj_ were again inserted into the ANCOVA as covariables, as we decided to use the residual ICV correction method for GM and WM volumes.

Finally, we ran Pearson’s correlation tests between ICVs, GM volumes, WM volumes, and each of the following demographic variables: age, years of schooling, and socioeconomic status. The GM and WM volume correlation analyses were repeated using partial correlation rates after employing the residual method.

## RESULTS

### Demographic Aspects

Among the 176 individuals analyzed, their ages varied between 66 and 80 years of age, and there were no significant differences between the ages of men and women (70.69±2.64 for women *vs.* 70.31±2.52 for men) (Table 1).

The male participants had significantly more years of schooling (3.89±3.13 *vs.* 5.42±4.13; *p*>0.01) and a higher socioeconomic status than women (Table 1). The years of schooling varied between 0 and 20 years, and there were participants from socioeconomic status levels A through F.

Having more years of schooling was positively associated with a higher socioeconomic status (*p*<0.001).

### Comparison of Brain Volumes Between Sexes

The ICV, WM volume, and GM volume values were significantly larger in men than in women (Table 2).

After ICV correction using the residual method, there was statistically significant evidence of larger WM and GM volumes in women.

Even after the inclusion of age and years of schooling as covariables in a separate ANCOVA analysis, the difference between the values of intracranial volume (ICV), WM volumes, GM volumes, gray matter volume after the residual correction method (GM_r_), and white matter volume after the residual correction method (WM_r_) remained statistically significant.

### Correlations

Pearson’s correlation results are shown in Table 3. The ICV value was directly associated with years of schooling (*p*=0.022) and socioeconomic status (*p*=0.002).

The GM values were also directly associated with years of schooling (*p*=0.022) and socioeconomic status (*p*=0.000); however, they were inversely associated with the participant’s age (*p*=0.042).

Regarding WM, we could only establish a statistically relevant relationship, a direct association between WM volume and socioeconomic status (*p*=0.022) (Table 3).

After the residual ICV correction method for GM and WM volumes was applied, there were no longer significant differences between brain volumes (GM_r_ and WM_r_) and age. However, there was still a significant difference, with a negative association between years of schooling and volumes of GM_r_ (*p*=0.040) and WM_r_ (*p*=0.020) and socioeconomic status, in addition to the values of GM_r_ (*p*=0.008) and WM_r_ (*p*=0.001) (Table 3).

## DISCUSSION

The present study demonstrated the presence of significant differences in neuroanatomical structure volumes between men and women in a population sample of participants above 66 years of age. Using raw volumes, we found larger GM and WM volumes and ICVs in men. However, after applying an ICV correction method based on partial brain volumes, we found larger relative volumes of GM and WM in women. Epidemiological data from the current study revealed a greater socioeconomic status and more years of schooling among the male population. This epidemiological difference was already expected in our participant sample, according to recent large population-based surveys in Brazil (24).

Our results are consistent with previous findings of larger brain volumes in men than in women before any adjustments. Barnes et al. (6) reported larger ICVs, GM volumes, and WM volumes in men than in women in the age range of 24 to 81 years. In a more recent study from Király et al. (4), the authors found larger subcortical structure volumes and larger partial brain volumes in men in a group of individuals aged between 21 and 58 years.

Currently, there are few studies with large samples that have evaluated brain volume variations between sexes during the aging process and have specifically investigated participants above 50 years of age. In most of these studies, the unadjusted brain volumes of GM and WM were found to be larger in men, as reported by Greenberg et al. (25), Voevodskaya et al. (26), and Pintzka et al. (3), and also in the present study.

Regarding WM, the results of the volume differences between sexes are, so far, discrepant among studies in the literature (27); therefore, it is not possible to establish a consistent pattern to which the results of the present study may be referred. Two of the largest studies carried out to date, respectively, by Greenberg et al. (25) and Pintzka et al. (3), showed that the WM volumes remained larger in men after adjustments for ICV using the proportion correction (that is, volume of interest/ICV). However, Voevodskaya et al. (26) and Pintzka et al. (3) showed that after ICV correction (using the proportions and residuals method, respectively), there was no longer a significant difference in WM volumes between men and women. Finally, in the previously mentioned study by Barnes et al. (6), the authors found larger volumes of WM in women after the ICV adjustment method was applied; this is similar to our findings obtained after applying the residual method.

Conversely, our findings of larger relative GM volumes in the female sample after the residual correction method was applied were consistent with several studies. The same result was observed by Pintzka et al. (3), Király et al. (4), Barnes et al. (6), Ikram et al. (13), and Greenberg et al. (25). In only one previous study, conducted by Voevodskaya et al. (26), differences in GM volumes between men and women were no longer significant after application of the proportions method to correct for ICV variations.

It is important to highlight that the chosen method for ICV correction has a direct implication on the results (12), since the choice affects the manner in which the ICV measurement errors are disseminated to the normalized volumes (28). The proportions method is implemented by creating a ratio between the partial neuroanatomical volume, such as the WM or GM volume, and the ICV (3,10,28,29). The ANCOVA method is performed by generating covariance analyses between the participant’s sex and neuroanatomical volume (WM or GM), in which the ICV value becomes a covariate (3,29). The proportions method is more susceptible to systematic errors due to the lack of proportionality between the ICVs and GM and WM volumes (3,28). The ANCOVA method can be flawed if the parameters included in the model (ICV and sex) are correlated. In that case, the ANCOVA method produces multiple results, equally valid, since both parameters could explain the GM and WM volume differences. Therefore, this method produces more than one valid result (10,30). For these reasons, we opted for a third ICV correction method, recently validated, called the residual method. Despite the limitations of needing samples of similar size and that are relatively comparable demographically (30), the residual method presents the advantages of being highly effective in removing ICV impact (3) and generating fewer systematic and random errors (10).

Even though the women in the current study had fewer years of schooling and a lower socioeconomic status overall, they had the largest volumes of GM after adjusting the data with the residual method. This result is consistent with many previous studies that have demonstrated a larger relative GM compartment volume in women than in men. Numerous studies point to a possible sex-dependent neuronal volume redistribution (8,31). The main hypothesis for the larger GM volume in women is that, since females have a smaller ICV, they would have developed a proportionally larger GM volume to compensate for the smaller number of total neurons, since GM has great procedural power (14). In general, this theory is well accepted; however, the main divergencies rely on its etiology: whether this compensation mechanism is dependent mostly on brain size or whether it is dependent on the sex of the person.

Another possible hypothesis, according to the findings of Ryan et al. (2), would be linked to sexual hormones. It is known that humans have estrogen receptors (ESR1 and ESR2) distributed throughout the brain, especially in limbic areas, which contribute to cognitive functioning (1,2,32) and emotional processing (1,33). Ryan et al. (2) has suggested that estrogen has a possible neuroprotective effect due to its influence on synaptic plasticity and its interaction with other neurotrophins. This type of effect could contribute to the larger GM volumes seen in women (2,34,35). Nevertheless, regarding the evaluation of the direct effect of estrogen on the human brain, the literature presents conflicting results, with data suggesting a detrimental effect of estrogen on brain volume in some studies (2), while a beneficial effect of hormonal therapy has been reported in other studies (1,34,36). On the other hand, specifically regarding the aging process, there are theories stating that the brain volume loss due to aging is not only greater but also starts earlier in men than in women (14,25,37-39). Thus, it is possible to conjecture that estrogen may play a protective role against brain atrophy, which could also explain why brain atrophy accelerates in women during the postmenopausal period. This theory could help explain the larger GM volumes found in elderly women after the ICV adjustment methods (14,23). Additional studies are necessary to fully explain the underlying mechanisms involved in the GM volume differences between sexes and to establish a causal relationship possibly involving genetics and/or hormonal influences that could affect this process (8).

Regarding the correlations between brain volumes and demographic variables, we did not observe significant relationships between age and any of the three main volumes of interest (ICV, GM after the residual correction method, and WM after the residual correction method). These negative results may be explained by the narrow age range of the present elderly sample. On the other hand, we found direct correlations between ICVs and years of schooling. This finding is relatively intriguing, given that previous studies have classified ICV as a measure of brain reserve rather than cognitive reserve (which has educational level as its main proxy) (40). A study by van Louenhoud et al. (40), however, pointed out a possible association between these two measurements in such a way that brain reserve would function as an intensifier and enhancer for cognitive reserve by allowing a greater adaptive potential for neurodegenerative processes. Similarly, various studies classify brain reserve as a more dynamic concept that can be related to cognitive reserve, which can also be subject to environmental influences via sensorial-cognitive stimuli (40,41). According to this recent framework, for example, years of education could prevent the loss of brain reserve and thus be associated with larger volumes of ICV in a population without any neurodegenerative diseases (40,42). It is worth highlighting that ICV was more significantly correlated with socioeconomic status, which is known to be interrelated with years of schooling. Further studies with representative samples of the population are warranted to evaluate the relationship between brain reserve and socioeconomic status throughout all stages of life in greater detail. The correlations in the opposite direction (that is, a larger volume linked to less education), found when we have used the relative volumes after the residual method (GM_r_ and WM_r_), are more difficult to interpret and have little support from the previous literature (15,16,18). Many findings suggest a positive relationship between years of schooling and larger volumes of GM in frontal and parietal regions (18), anterior cortical regions (16), and in the regions of the superior temporal gyrus, insular cortex, and cingulate gyrus (15). We interpreted the identified pattern in the present study as a consequence of the statistical correction method, since this method is ICV-dependent, and ICV was shown to be a variable directly correlated with years of schooling and socioeconomic status in our study.

One important limitation of the present study is the risk of systematic errors associated with the use of methods for automatic segmentation of brain volumes (23). Analyses of brain volumes as a whole have additional limitations, given that brain structures may present similar volumes and yet have distinct shapes. Moreover, by establishing an ICV correction method for brain volumes, we affirm that there is a more valid relationship between the anatomical and functional description of the structures and their relative volumes than between the anatomical and functional description of the structures and their absolute volumes. Nonetheless, there are studies that indicate a strong relationship between the absolute brain volume and key cognitive functions (3). One final limitation of our study is the use of years of schooling to estimate educational attainment, since individuals with similar years of attendance at school can have different levels of dedication to studying and cognitive enrichment throughout life.

In conclusion, in a population sample of 176 elderly individuals, including those with relatively low educational levels and without any neurodegenerative diseases, we demonstrated differences in overall brain volumes between men and women. This difference was maintained even after the correction of GM and WM volumes for the total ICV, which reveals that part of the dimorphism between sexes can be attributed to the characteristics of each sex and not merely to a difference in cephalic size. We have identified larger volumes of GM in women, even though this particular group has had the lowest levels of socioeconomic status and fewer years of education. Further studies evaluating hormonal and molecular genetic aspects are needed to explain why women, despite their smaller cranial size, present larger relative GM volumes than those of men, and to explain how this process occurs.

## AUTHOR CONTRIBUTIONS

Busatto GF conceived the study, participated in study design, interpreted the data and revised the manuscript. Buchpiguel M analyzed and interpreted the data, drafted and revised the manuscript. Squarzoni P, Tamashiro-Duran JH, Duran FL, Leite CC, Scazufca M, Lotufo PA, and Alves TC interpreted the data and revised the manuscript. All authors have read and approved the final version of the manuscript.

## Figures and Tables

**Table 1:** Demographic aspects in the sample.

		Total Sample(n=176)	Women(n=92)	Men(n=84)	*p*
Age (SD)		70.5114	70.6957±2.643	70.3095±2.522	0.323
Years of Schooling (SD)		4.6193	3.8913±3.132	5.4167±4.128	0.007*
Socioeconomic Status	A	5	1	4	0.02*
	B	37	12	25	
	C	93	51	42	
	D	36	25	11	
	E	4	2	2	
	F	1	1	0	

*p<0.05, statistically significant. SD - Standard-deviation.

**Table 2:** Intracranial, WM, and GM volumes before and after the use of ICV correction method.

		Total Sample	Women	Men	*p*
ICV (SD)		1351842.000	1275499.913±112706.472	1433613.571±126214.227	<0.001*
GM (SD)		514003.494	491284.859±33982.461	538486.083±42942.107	<0.001*
WM (SD)		407018.580	383344.380±45503.976	433504.726±45129.32549	<0.001*
Residuals Correction Method (SD)	GM_r_		556711.355±71897.443	466828.038±79399.760	<0.001*
	WM_r_		447412.541±66333.789	363334.391±75226.867	<0.001*

*p<0.05, statistically significant. SD - Standard-deviation.

GM_r_: gray matter volume after the residual correction method; WM_r_: white matter volume after the residual correction method; ICV: intracranial volume.

**Table 3:** Correlations between age, years of schooling, and socioeconomic status and ICV and GM, WM, GMr, and WMr volumes (before and after the correction using the residual method).

		Age	Years of Schooling	Socioeconomic Status
ICV	Pearson’s Correlation	-0.097	0.172	0.235
	Sig. (2 tailed)	0.198	0.022*	0.002*
GM	Pearson’s Correlation	-0.154	0.172	0.261
	Sig. (2 tailed)	0.042*	0.022*	0.000*
WM	Pearson’s Correlation	-0.121	0.127	0.173
	Sig. (2 tailed)	0.109	0.094	0.022*
GM_r_	Pearson’s Correlation	0.059	-0.155	-0.198
	Sig. (2 tailed)	0.440	0.040*	0.008*
WM_r_	Pearson’s Correlation	0.068	-0.175	-0.239
	Sig. (2 tailed)	0.373	0.020*	0.001*

*p<0.05 - statistically significant.

GM_r_: gray matter volume after the residual correction method; WM_r_: white matter volume after the residual correction method; ICV: intracranial volume.
